# Correction: Fluorescence detection of three types of pollutants based on fluorescence resonance energy transfer and its comparison with colorimetric detection

**DOI:** 10.1039/d4ra90033b

**Published:** 2024-04-08

**Authors:** Yifei Kong, Dan Liu, Xinran Guo, Xinyue Chen

**Affiliations:** a School of Pharmacy, Lanzhou University Lanzhou 730000 P. R. China chenxinyue888@126.com +86-15293109642

## Abstract

Correction for ‘Fluorescence detection of three types of pollutants based on fluorescence resonance energy transfer and its comparison with colorimetric detection’ by Yifei Kong *et al.*, *RSC Adv.*, 2023, **13**, 22043–22053, https://doi.org/10.1039/D3RA02647G.

The authors regret that an incorrect version of Fig. 1A was included in the original article. The correct version of Fig. 1 is presented below.

**Fig. 1:**
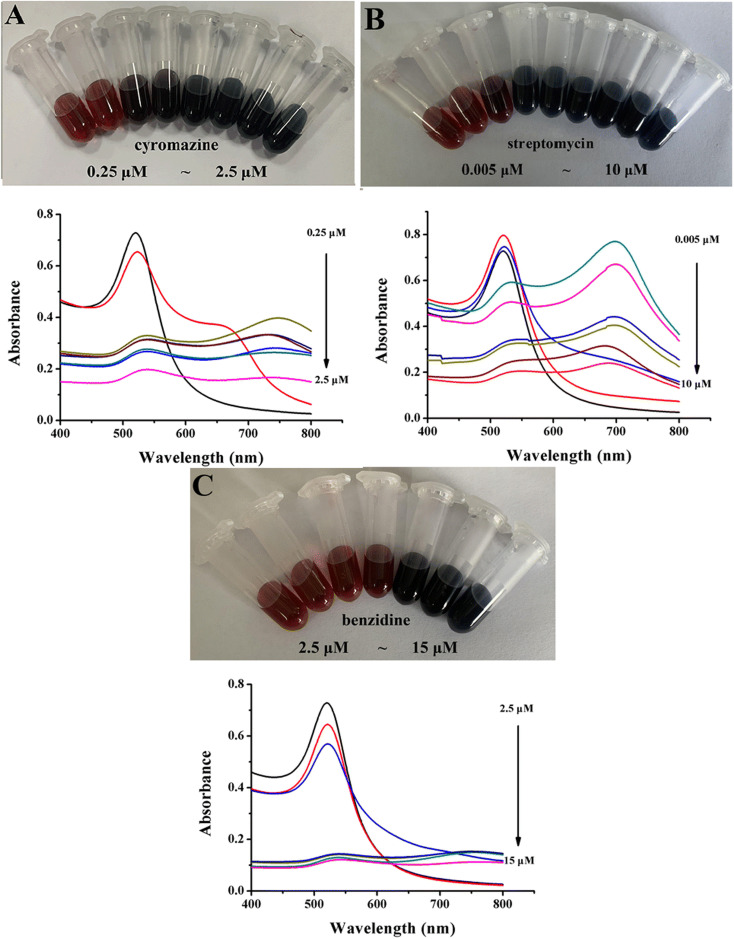
Photograph and UV-vis absorption spectra of AuNPs after the addition of various concentrations of cyromazine (A), streptomycin (B), and benzidine (C).

The Royal Society of Chemistry apologises for these errors and any consequent inconvenience to authors and readers.

## Supplementary Material

